# Indexing amyloid peptide diffraction from serial femtosecond crystallography: new algorithms for sparse patterns

**DOI:** 10.1107/S1399004714026145

**Published:** 2015-01-23

**Authors:** Aaron S. Brewster, Michael R. Sawaya, Jose Rodriguez, Johan Hattne, Nathaniel Echols, Heather T. McFarlane, Duilio Cascio, Paul D. Adams, David S. Eisenberg, Nicholas K. Sauter

**Affiliations:** a Physical Biosciences Division, Lawrence Berkeley National Laboratory, Berkeley, CA 94720, USA; b UCLA–DOE Institute for Genomics and Proteomics, University of California, Los Angeles, CA 90095-1570, USA; c Department of Biological Chemistry, University of California, Los Angeles, CA 90095-1570, USA; d Howard Hughes Medical Institute, University of California, Los Angeles, CA 90095-1570, USA; e Department of Bioengineering, University of California, Berkeley, CA 94720, USA

**Keywords:** XFEL, Sup35 yeast prion, indexing methods, crystallography

## Abstract

Still diffraction patterns from peptide nanocrystals with small unit cells are challenging to index using conventional methods owing to the limited number of spots and the lack of crystal orientation information for individual images. New indexing algorithms have been developed as part of the *Computational Crystallography Toolbox* (*cctbx*) to overcome these challenges. Accurate unit-cell information derived from an aggregate data set from thousands of diffraction patterns can be used to determine a crystal orientation matrix for individual images with as few as five reflections. These algorithms are potentially applicable not only to amyloid peptides but also to any set of diffraction patterns with sparse properties, such as low-resolution virus structures or high-throughput screening of still images captured by raster-scanning at synchrotron sources. As a proof of concept for this technique, successful integration of X-ray free-electron laser (XFEL) data to 2.5 Å resolution for the amyloid segment GNNQQNY from the Sup35 yeast prion is presented.

## Abstract 2

Special methods are required to interpret sparse diffraction patterns collected from peptide crystals at X-ray free-electron lasers. Bragg spots can be indexed from composite-image powder rings, with crystal orientations then deduced from a very limited number of spot positions.

## 1. Introduction   

Automated indexing of crystallographic diffraction patterns from protein samples is a critical first step in the data reduction necessary to derive atomic coordinates from measured reflection intensities. The Rossmann indexing algorithm (Steller *et al.*, 1997 ▶), as implemented in *MOSFLM* (Powell, 1999 ▶) and *LABELIT* (Sauter *et al.*, 2004 ▶), is robust for most problems encountered in protein crystallography. However, some images do not contain enough spots to identify the periodicity needed to discover the reciprocal basis vectors using Fourier analysis. These sparse patterns, either as a consequence of being exceptionally low resolution or having exceptionally small unit-cell dimensions, are difficult to analyze using previously described methods.

The difficulty in analyzing these crystals is exacerbated when the data are not collected using the single-crystal rotation method but are collected in the form of still images from randomly oriented crystals, such as when using the serial crystallography technique typically employed at XFEL sources. Here, the exposure is too short to allow sufficient crystal rotation that would bring additional reflections into diffracting conditions. In the absence of any prior information about the crystal, nine parameters are determined during traditional indexing: six for the unit-cell parameters and three for the crystal orientation. We have generally found that a minimum of 16 reflections are necessary to index XFEL images produced by macromolecular crystals (Hattne *et al.*, 2014 ▶), and that the outcome is improved if the approximate unit-cell parameters are known ahead of time to act as a constraint (Gildea *et al.*, 2014 ▶). However, sparse XFEL patterns can have fewer than 16 spots per image, can lack obvious periodicity, can have multiple lattices per image and can have other spot pathologies such as streakiness and splitting. These issues are not necessarily limited to XFEL sources and make new techniques for indexing sparse patterns desirable. While a compressive sensing technique has been proposed that could in theory index sparse images (Maia *et al.*, 2011 ▶), it has not yet been applied to experimental data, and we additionally sought to develop a method that can use prior knowledge of the unit cell.

**Figure 1:**
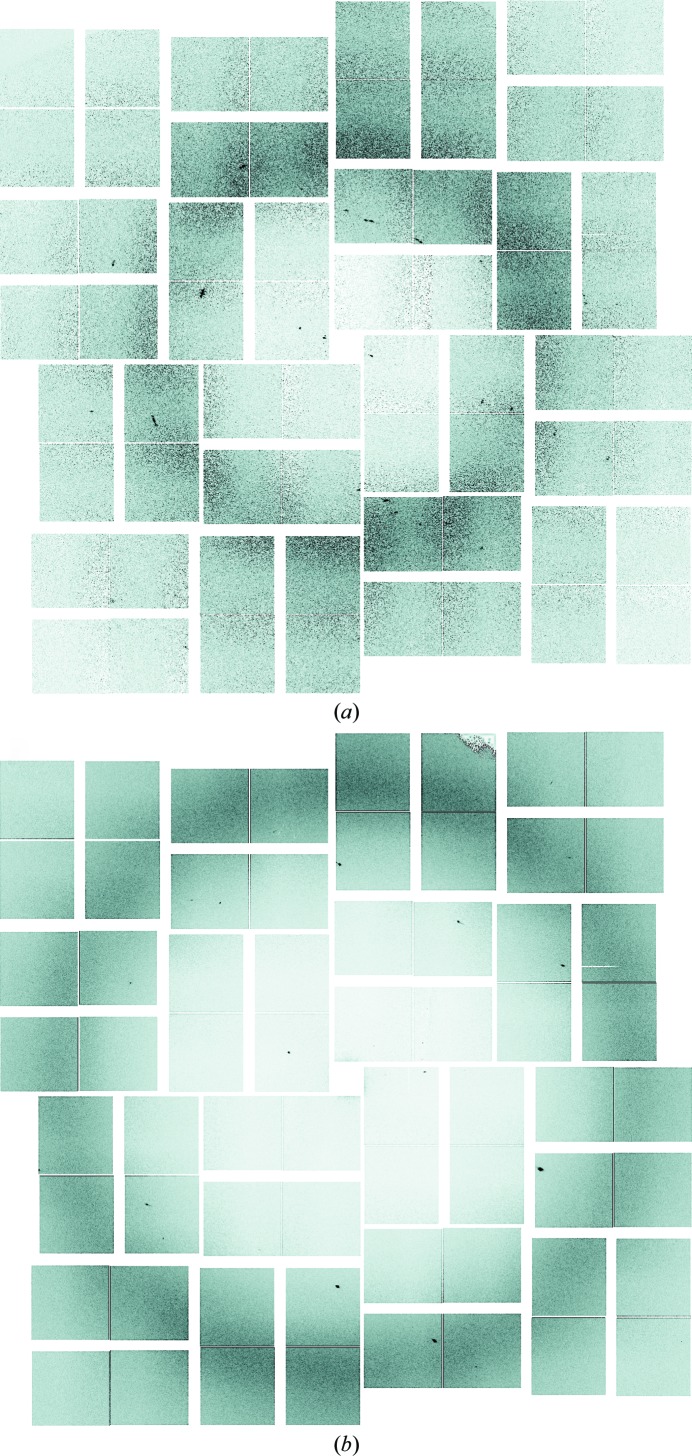
Example GNNQQNY diffraction patterns at different detector distances (111 and 166 mm). (*a*) One of the clearer GNNQQNY images, with obvious periodicity. Note the spot pathologies, including split spots and streaked spots. (*b*) Typical GNNQQNY image with few spots visible. Both (*a*) and (*b*) are indexable with *cctbx.small_cell.*

As a test case, we investigated sparse data from amyloid peptide crystals collected using the XFEL at the Linac Coherent Light Source (LCLS; Fig. 1 ▶). Amyloid-like fibrils are associated with many human diseases, such as Alzheimer’s disease, Parkinson’s disease, type II diabetes, amyotrophic lateral sclerosis (ALS) and dialysis-related amyloidosis. The fibrils consist of partially unfolded proteins which self-associate through a short segment comprising the ‘cross-β spine’ (Sipe & Cohen, 2000 ▶). Understanding the structural packing of these fibrils is critical to the development of clinical treatments. To this end, the yeast protein Sup35 has been studied as a model system owing to its prion-like properties (Wickner, 1994 ▶; Patino *et al.*, 1996 ▶; Serio *et al.*, 2000 ▶; King & Diaz-Avalos, 2004 ▶; Tanaka *et al.*, 2004 ▶). Sup35 contains a seven-amino-acid sequence GNNQQNY that when isolated displays the fibril-like formation of the full-length protein (Balbirnie *et al.*, 2001 ▶). Its structure has been solved from a single microcrystal at a microfocus synchrotron beamline (Nelson *et al.*, 2005 ▶).

Since the structure of GNNQQNY has been solved at relatively high resolution from rotation data collected at a synchrotron source, it was an ideal test for new indexing methods utilizing still images. Microcrystals of GNNQQNY were examined by flowing crystals across the XFEL beam. The crystals were destroyed as they intersected X-ray pulses, but single diffraction images were collected before damage was accrued. To index these images, we developed *cctbx.small_cell*, a new indexing program within the open-source *Computational Crystallography Toolbox* (*cctbx*) package (Grosse-Kunstleve *et al.*, 2002 ▶; Sauter *et al.*, 2013 ▶).

## 2. Materials and methods   

### 2.1. Sample preparation   

Lyophilized synthetic GNNQQNY (AnaSpec, CS Bio) peptide dissolves easily in water and aqueous solutions. GNNQQNY was dissolved in pure water (resistivity = 18.2 MΩ cm) at 10 mg ml^−1^ and filtered through a 0.22 µm filter. Initial crystals were grown by hanging-drop diffusion (5 and 10 µl drops) with 1 *M* NaCl in the reservoir. These initial crystals were used as seeds for bulk crystallization. Bulk crystallization was performed with a 500 µl solution of 10 mg ml^−1^ GNNQQNY dissolved in water and filtered. Seeds for bulk crystallization were made by vortexing the hanging-drop crystals with a flamed glass rod for ∼60 s, creating a ‘seed solution’. 10 µl of the seed solution was added to the 500 µl GNNQQNY solution to accelerate crystallization. Crystals grew in 2–3 d at 20°C. To prepare the crystals for the liquid injector, the crystals in the bulk crystallization solutions were vortexed with a flamed glass rod for ∼20 s to break up crystal clusters. These crystals were subsequently filtered through a 10 µm filter prior to diffraction experiments. For injection, we prepared 1 ml of slurry (25 µl of crystal pellet suspended in 1 ml of water).

### 2.2. Data collection   

Needle crystals 20 µm long and 2 µm thick of the peptide GNNQQNY were injected using a microinjection system (Weierstall *et al.*, 2012 ▶) at the Coherent X-ray Imaging (CXI) instrument of LCLS over the course of 21.3 min. The X-ray source was configured using a 1 µm beam focus, with an X-ray wavelength of 1.457 Å. The sample chamber was at room temperature under vacuum. The *Spotfinder* algorithm (Zhang *et al.*, 2006 ▶) could detect spots in 8704 of the 152 752 serial XFEL images using the default spotfinding parameters (which are very permissive).

### 2.3. Determining unit-cell parameters using a composite powder pattern   

**Figure 2:**
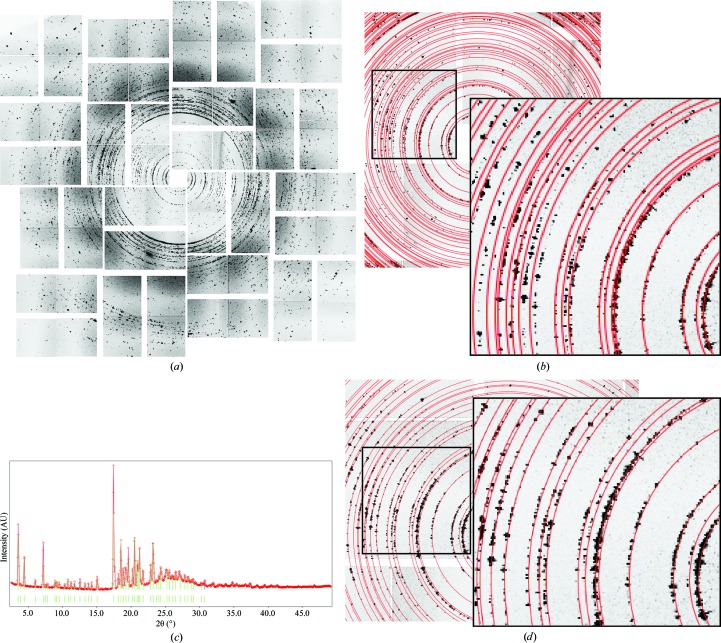
Derivation of unit-cell parameters from a powder pattern. (*a*) GNNQQNY maximum-value composite image from 32 178 diffraction patterns. Powder rings are visible in the composite. (*b*) Unit cell from the published GNNQQNY structure (PDB entry 1yjp). Calculated powder rings are overlaid in red. (*c*) Radial averaging trace from the composite pattern displayed in *Rex.cell*. Peaks used for indexing are marked in green. (*d*) As (*b*) with the corrected *Rex.cell*-derived unit cell.

The GNNQQNY structure has been solved using synchrotron radiation (PDB entry 1yjp; Nelson *et al.*, 2005 ▶) using similar crystallization conditions as used in this study. The crystals belonged to space group *P*2_1_, with unit-cell parameters *a* = 21.94, *b* = 4.87, *c* = 23.48 Å, β = 107.08°. In order to determine whether our preparation of crystals had an identical unit cell, we created a ‘maximum-value’ composite diffraction image of a portion of the total data. XFEL data collection is typically subdivided into slices known as ‘runs’, where each run is 5–10 min of data-collection time, typically at 120 Hz. As not all data were sampled at the same detector distance, we created composites from each run and selected the composite with the most signal: 32 178 images at a constant detector distance and wavelength (Fig. 2 ▶ *a*). This composite simulates a powder diffraction image by assigning the intensity of each pixel to be the maximum value recorded at that pixel throughout the data set. This is performed without filtering out any images based on signal intensity to guarantee the sampling of even weak data. This method offers an advantage over an averaged image in that the powder rings appear sharper. We then overlaid the predicted powder rings from the 1yjp unit-cell parameters (Fig. 2 ▶ *b*). We found that the predicted rings did not align with the maximum-value composite, even after slight adjustments to the detector distance or wavelength that would increase or shrink the predicted pattern, indicating that the unit-cell parameters needed adjustment.

To determine the actual unit-cell parameters, we calculated a radial average of the maximum-value composite, as has been performed previously for amyloid micro-crystal powder diffraction (Sunde & Blake, 1998 ▶; Balbirnie *et al.*, 2001 ▶; Diaz-Avalos *et al.*, 2003 ▶; Makin *et al.*, 2005 ▶). We processed the radial average using *Rex.cell*, a freely available software package designed to index powder diffraction patterns (Fig. 2 ▶ *c*; Bortolotti & Lonardelli, 2013 ▶). After peak finding, *Rex.cell* was able to index the radial average using the *N-TREOR* algorithm (Altomare *et al.*, 2009 ▶), resulting in the corrected *P*2_1_ unit-cell parameters *a* = 22.23, *b* = 4.86, *c* = 24.15 Å, β = 107.32° (Fig. 2 ▶ *d*). Note that while the unit-cell parameters are similar to the published result, the small differences translate into large changes in the radii of the predicted powder rings. The original 1yjp structure was solved from a crystal that had dried on the surface of a capillary, while the XFEL crystals were fully hydrated; this could account for the small differences in unit-cell parameters.

We estimated the standard deviation (σ) of the powder pattern-derived unit-cell lengths to be on the order of 1%. To estimate this, we generated a large population of model unit cells (10 000) varying in the *a* and *c* dimensions but otherwise identical to the powder pattern-derived unit-cell parameters. The *a* and *c* values were modeled with Gaussian distributions, with means centered on the *Rex.cell* *a* and *c* values and standard deviations σ_model,*a*_ and σ_model,*b*_, respectively. This population of models was used to compute diffraction angles (2θ) for four low-resolution reflections [(1, 0, 0), (−1, 0, 1), (1, 0, 1) and (2, 0, 0)]. Histograms of these 2θ angles were compared with the experimentally determined radial average profile from our composite powder pattern. This procedure was repeated for several σ_model_ values in order to match the histogram peak widths with the measured peak widths. Higher-resolution Miller indices were not amenable to this analysis since the corresponding peaks in the composite radial average were distorted (broadened) by uncertainties in sensor positions and overlap with neighboring powder rings. For this reason, it was not possible to estimate the standard deviation of the *b* axis.

### 2.4. *cctbx.small_cell*: a new program for indexing peptide XFEL diffraction data   

**Figure 3:**
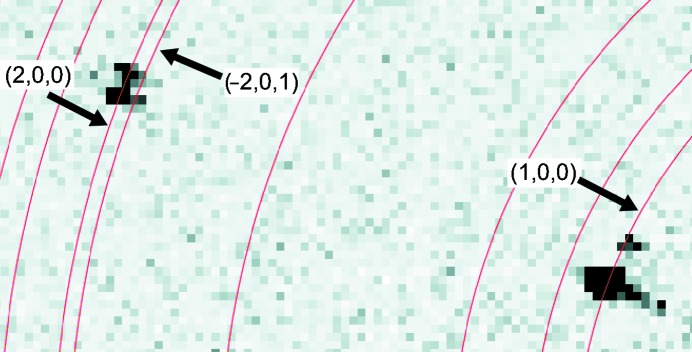
Indexing a single still shot. Two spots are shown from Fig. 1 ▶(*a*). Predicted powder rings are overlaid in red. Rings that overlap a spot represent potential Miller indices for that spot. The index of the spot in the upper left corner is ambiguous owing to its proximity to two closely spaced powder rings. The pair of spots in the lower right corner illustrates an ambiguity likely owing to crystal splitting.

Once we had derived accurate unit-cell parameters, we developed a new program capable of processing this difficult data set. Given a known set of crystal and experimental parameters (unit cell, detector distance from the crystal, incident beam energy and beam center on the image), the distance between the beam center on an image and a given reflection will correspond to one or more known reciprocal-space *d*-spacings from a predicted powder pattern (Fig. 3 ▶). Therefore, for each individual image the indexing algorithm involves three main steps: (i) assign initial Miller indices to the reflections based on the model powder pattern, (ii) resolve indexing ambiguities that arise from closely clustered powder rings and from the symmetry of the crystal’s lattice and (iii) calculate basis vectors and refine the crystal orientation matrix. After these three steps have been performed, spot prediction, integration and merging proceeds as implemented in other packages, with some exceptions.

### 2.5. Resolving indexing ambiguities using a maximum-clique algorithm   

Determining which powder ring a reflection overlaps is not sufficient to assign its unique Miller index owing to ambiguities that arise from several sources: errors in detector position, wavelength and beam center, multiple possible powder rings overlapping the same reflection and, most importantly, symmetry. These ambiguities can be divided into four types. The first is the most straightforward: reflections often intersect two or more closely clustered powder rings (Fig. 3 ▶). This effect is most pronounced for high diffraction angles or large unit-cell parameters. The second ambiguity arises from the need to determine which lattice symmetry operator maps the reflection to the asymmetric unit. For example, in addition to the identity operator (*h*, *k*, *l*) and the Friedel operator (−*h*, −*k*, −*l*), the reciprocal lattice of the GNNQQNY crystals has a twofold symmetry axis with the operator (−*h*, *k*, −*l*). Combining the Friedel symmetry with the twofold symmetry operator yields a fourth symmetry operator (*h*, −*k*, *l*), which completes the lattice group. For any given Bravais lattice, there will exist a list of symmetry operators that generate the complete set of Miller indices from the asymmetric unit.

Given any set of observed reflections, one of them may arbitrarily be selected as the reference reflection residing in the asymmetric unit, and for all others the relative symmetry operation must be determined. It is only when multiple reflections are examined together that these first two ambiguities can be resolved.

**Figure 4:**
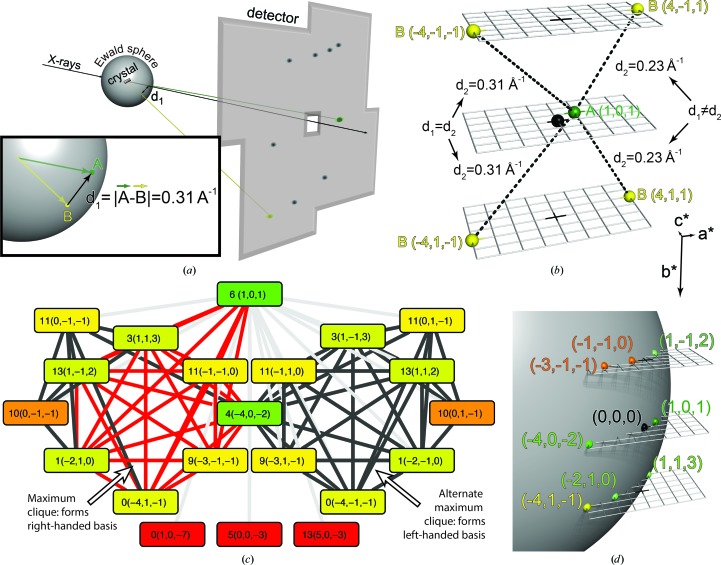
Resolving indexing ambiguities in the diffraction pattern from Fig. 1 ▶(*b*) using a maximum clique. (*a*) Calculation of *d*_1_, the observed distance in reciprocal space between two reflections. A reference reflection *A* and a candidate reflection *B* are projected back on to the Ewald sphere from their positions on the detector. Inset: the distance between the reflections *A* and *B* is measured in reciprocal space. (*b*) Calculation of *d*_2_, the predicted distance in reciprocal space. Given the reference reflection *A* and its candidate index (1, 0, 1), there are four possible symmetry operators applicable to reflection *B* and its candidate index (4, 1, 1). Two of them are not correct, as the predicted distances *d*_2_ do not match the observed distance *d*_1_. (*c*) Complete graph from Fig. 1 ▶(*b*). Each node represents a single reflection paired with a candidate Miller index and one of four symmetry operators of the reciprocal-lattice point group. The boxes are labeled first with an arbitrary identification of the spot (a spot ID) and then with the Miller index being examined. For example, the central spot is spot number 4, with index (−4, 0, −2). The nodes are colored by degree (number of connections), with green representing many connections and red representing one. Edges represent spot connections (see text). (*d*) Plotting the eight reflections from the correct maximum clique in (*c*) in reciprocal space. The plotted reflections form a right-handed basis and intersect the Ewald sphere.

Imagine the case where potential Miller indices **h**_*A*_ and **h**_*B*_ for two measured reflections *A* and *B* have been assigned based on overlap of their powder rings. The goal is to determine whether the indices are correct, and if they are, to determine the symmetry operator **w**_*BA*_ moving *B* into the same asymmetric unit as *A*. We can measure the reciprocal-space distance between the spots by calculating their three-dimensional reciprocal-space positions **x**_*A*_ and **x**_*B*_ (using the experiment’s detector geometry and wavelength), and calculating the magnitude of the displacement
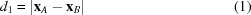
between them (Fig. 4 ▶ *a*). Here, we assume that the reflections are exactly on the Ewald sphere; their location in reciprocal space is determined only by the pixel coordinates of the spot centroid. Issues that can lead to the reflection not being located precisely on the Ewald sphere, for example partiality inherent in still exposures, crystal mosaicity or a non-monochromatic incident beam, are ignored. We can also predict the reciprocal-space distance between the two candidate Miller indices as follows. Under the assumption that we have correctly identified the Miller indices and relative symmetry operator, the Miller index difference between the two reflections is
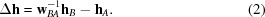



The unit-cell parameters can be expressed in a rotation-independent manner in the form of a metrical matrix
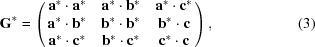
where a*, b* and c* are the reciprocal-space basis vectors. The metrical matrix gives us the distance between two reflections,
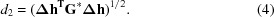



The observed distance *d*_1_ is then compared with the predicted distance *d*_2_ under each possible lattice symmetry operation that could relate the two reflections (Fig. 4 ▶ *b*). If the two distances match within a given tolerance, then it is provisionally concluded that the candidate indices are correct, that the reflections are on the same lattice and that the mutual symmetry operation is correct. Ideally, only one symmetry operation will yield a predicted distance *d*_2_ that matches the observed distance *d*_1_.

However, sometimes two symmetry operators yield the same predicted distance *d*_2_, leading to the third type of indexing ambiguity that *cctbx.small_cell* needs to resolve. Imagine again two spots *A* and *B*, but this time *A* is a centric reflection and *B* is a noncentric reflection. In this case, two possible symmetry operators will give the same, correct value of *d*_2_. However, if a third, noncentric reflection is introduced into the system, mutual comparison among all three reflections can resolve the ambiguity.

*cctbx.small_cell* resolves these three types of indexing ambiguities simultaneously by treating the set of reflections on the image as a graph where the nodes are all potential combinations of indices **h** and symmetry operators **w** for each spot. For example, in *P*2_1_ each spot will be represented in the graph by four nodes, one for each of the four symmetry-related Miller indices sharing the same Bragg spacing (*i.e.* powder rings sharing identical radii). If a spot overlaps two powder rings, there will be eight corresponding graph nodes. None of the nodes arising from a single given spot are allowed to be connected to each other. Edges that connect nodes are drawn when (1) and (4) yield matching distances between observed and predicted reciprocal-space distances.

After building this graph, the reference spot is defined to be the most highly connected node. No symmetry operator will be assigned to it (or rather, its symmetry operator is the identity matrix). In the case where multiple spots have the same number of connections, the tie is resolved by choosing the spot whose connections are on average ‘shortest’. In other words, if the length of an edge is defined as the difference between the measured and predicted locations in reciprocal space (Δ*d* = |*d*_1_ − *d*_2_|), then the reference spot is the highest connected spot with the shortest connections on average.

At this point we narrow the graph to the set of spots that are connected to the reference spot. A pivoting Bron–Kerbosch algorithm is applied to determine the maximum clique in the graph, *i.e.* the largest set of nodes in the graph that are all connected to each other. For more information, see Cazals & Karande (2008 ▶) and Appendix *A* [App appa].

When this is complete, a clique of spots has been determined with completely resolved indices and symmetry operators. Each spot will occur in the maximum clique exactly once, resolving the first, second and third types of ambiguity. A visual example of an actual maximum clique produced by *cctbx.small_cell* to index the pattern in Fig. 1 ▶(*b*) is shown in Fig. 4 ▶(*c*). In this example, spot 6 (an arbitrary identifier) was chosen as the reference spot with index (1, 0, 1). The reference spot is connected to all of the spots in the figure; its edges are shown in a lighter gray. Edges connecting the seven-node maximum clique plus the reference spot are in red. This graph contains a centric reflection (spot 4) and two alternate ways of indexing the other spots (left and right halves of the graph). A second maximum clique exists on the right half of the graph, with the same spots as the left but with a different choice of symmetry operations. The choice between them appears to be arbitrary, but closer examination reveals that the right half corresponds to a left-handed basis and thus is readily rejected. Note that within the chosen clique the indexing is consistent. For example, if spot 3 is indexed as (1, 1, 3) spot 1 cannot be (−2, −1, 0); it must be (−2, 1, 0). Also note that spot 11 overlaps two powder rings, and thus appears four times in this graph: twice in each half. Owing to index (−1, −1, 0) being more connected than (0, −1, −1), the former is chosen as its index. Finally, at the bottom of the panel there are three candidates that are not connected to any of the other nodes except for the reference spot. Spots 0 and 13 turn out to be alternate indices from overlapping powder rings connected within the given tolerance to spot 6 but not to any of the other spots in the graph. Spot 5 appears to be in a secondary lattice (not shown). Fig. 4 ▶(*d*) plots the maximum clique in reciprocal space, revealing how it conforms to the Ewald sphere.

We also note here that the technique as presented will be more difficult to utilize for triclinic cells. In addition to being difficult to index from powder patterns, the derived crystal orientation will be less accurate for these crystal systems owing to the lack of symmetry restraints to guide refinement.

### 2.6. Overcoming diffraction pathologies arising from crystal disorder   

Two kinds of diffraction pathologies arising from crystal disorder are directly treated by *cctbx.small_cell*. Firstly, spots that are obviously too large (more than 100 pixels) or that are extended in the radial or azimuthal directions are discarded. This phenomenon is common in data sets of small peptides (Fig. 1 ▶ *a*). Secondly, a fourth type of ambiguity can occur wherein two spots are assigned the same index after the resolution of the maximum clique. This can happen when the measured locations of the two spots in reciprocal space are very close, within the threshold being used for determining connectivity. This is likely to be caused by split spots, multiple lattices or other pathologies. We resolve this final type of ambiguity by finding which spot among those with the same index has the shortest average connections and then removing the other spots with the same index from the maximum clique, similar to how we resolve ties in determining the reference spot.

### 2.7. Using the maximum clique to derive basis vectors   

For the *i*th reflection with Miller index **h**_*i*_ and symmetry position **w** ^−1^_*i*,ref_ **h**_*i*_ (the subscript ‘ref’ signifies that we have assigned one spot as the reference for determination of the asymmetric unit), the observed reciprocal-space position **x**_*i*_ is given by
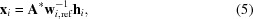
where A* = [a*b*c*] is the crystal orientation matrix consisting of the reciprocal-space basis vectors. The crystal orientation, which is initially unknown, is determined by solving for A* over *n* equations, one for each reflection. The system is overdetermined for *n* > 3; thus, we use a linear least-squares approach implemented in the package *Numpy* (Walt *et al.*, 2011 ▶). In practice, we require *n* to be at least 5, based on examining the accuracy of the derived orientation matrices. This was performed visually by comparing the locations of the predicted reflections and their associated observations for images where *n* was 3 or 4.

The accuracy of our maximum-clique indexing will directly affect the quality of the basis vectors derived from these equations. For example, as noted above, spot 11 from Fig. 4 ▶(*c*) overlaps two powder rings. While ambiguous reflections such as these could in theory be ignored until the proper orientation is determined, at which point their correct index would be obvious, in practice most reflections overlap multiple powder rings, especially at higher resolution, so both possibilities must be considered. Hence, if only reflections clearly overlapping a single ring are used there would be too few data points for the maximum-clique technique to succeed. Further, here we see how including these ambiguous reflections in the complete graph and solving the maximum clique improves the results. When the highest connected index for reflection 11 is chosen, (−1, −1, 0), the unit-cell parameters derived from solving the above equations are closer to the known cell from the powder pattern than when using the less connected index (0, −1, −1) (see Table 1 ▶). The correct index is likely to be that derived from the more connected clique.

### 2.8. Refining the crystal orientation matrix, reflection prediction, integration and structure solution   

Two criteria can be used to measure the success of the algorithm: (i) are the unit-cell parameters the same as the parameters derived from the composite powder pattern and (ii) does the crystal orientation matrix provide a model that successfully predicts reflection locations? We found that the first criterion depended on the number of indexed reflections (Supplementary Fig. S1). As the number of indexed reflections increased, the unit-cell parameters derived from (5) more closely matched the cell obtained from *Rex.cell*. Or, put differently, images with fewer indexed reflections yielded more divergent unit-cell parameters. We wanted to develop a refinement routine that could improve the accuracy for these more sparse patterns. To this end, after calculating basis vectors from (3), we extracted Euler angles and further refined them against the observed reflections using a simplex minimizer (Nelder & Mead, 1965 ▶) and the target function 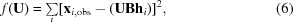
where **U** and **B** are the rotational and orthogonalization components of the reciprocal matrix A* (A* = **UB** = [a*b*c*]), respectively (Busing & Levy, 1967 ▶). Here, the sum squared difference between the observed position in reciprocal space of a spot (**x**_*i*,obs_) and its predicted position given its Miller index **h**_i_ and an input rotation matrix **U** is minimized over all *n* spots. **B** is determined by the powder pattern unit-cell parameters and is held constant; only the pure rotation **U** is refined. After refinement, spot locations are predicted again, giving us the second measure of the success of the algorithm, namely that new spots are found that can be indexed based on the predictions.

We then iterate, adding spots to the clique that had previously been rejected if they lie within a certain distance of a prediction on the detector, regenerating the basis vectors using this new clique and re-refining the orientation matrix. The iterative process is complete when we can add no more spots to the clique. We then integrate the indexed spots according to standard methods (Leslie, 2006 ▶). Presently, we only integrate the bright reflections from *Spotfinder*. It is not possible to integrate predicted reflections directly for two reasons: firstly, we do not yet have a good model for mosaicity which would enable us to determine which reflections are in the diffracting condition. Secondly, the orientation matrices that we generate, while accurate enough to produce the data presented here, do not provide sufficient precision to be confident that noise is not being integrated instead of signal.

The run time of the program is on the order of 3–10 s per image. Presently, multiple lattices are not used, although the algorithm could in principle find multiple unconnected cliques in a graph and thus identify multiple lattices. Roughly 50% of the GNNQQNY crystals exhibit split pathologies (Supplementary Fig. S2).

Merging was performed using *SCALEPACK* (Otwinowski & Minor, 1997 ▶) without attempting to put the images on a consistent scale. Scaling of XFEL data is a matter of ongoing research, and was not attempted here beyond the simplistic Monte Carlo approach (Kirian *et al.*, 2010 ▶) that averages all intensity measurements for a given Miller index. Molecular replacement (MR) was performed using PDB entry 1yjp as the search model with *Phaser* (see Table 2 ▶; McCoy *et al.*, 2007 ▶). The MR solution had LLG and TFZ scores of 34 and 5, respectively.

## 3. Results   

Of the 8704 images identified by *Spotfinder* to contain possible signal, 232 could be indexed with the current version of *cctbx.small_cell*. The permissive spotfinding settings help us to eliminate false negatives, but give many false positives. Of the 8472 that did not index, 3971 had zero spots in the maximum clique, indicating that the spots found were not on powder rings (noise or pathological spots). 4074 had maximum cliques small enough that the basis-vector calculation failed (usually 2–3 spots in total, indicating the diffraction on the image was weak or pathological). The remaining 427 had fewer than five total integrated spots and so were ignored (see also Supplementary Fig. S3).

Basis vectors derived from solving (5) for the 232 indexed images were averaged to determine a derived set of unit-cell parameters (Table 2 ▶). The standard deviations of the population of unit-cell lengths derived from each of the 232 indexed images are around an order of magnitude greater than the estimated 1% error in the unit-cell lengths derived from powder pattern indexing. This indicates that the majority of the error in the maximum-clique technique is likely to come from other sources than the powder pattern itself. It is likely that the number of reflections in the maximum clique is the largest contributor (Supplementary Fig. S1).

**Figure 5:**
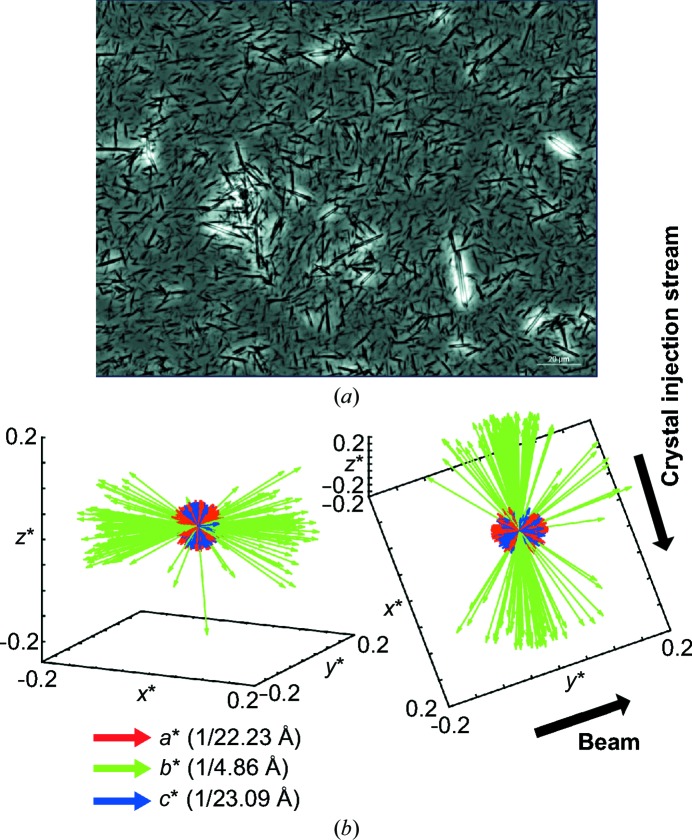
GNNQQNY needle crystals preferentially orient in the sample-delivery stream. (*a*) Optical microscope image of GNNQQNY needle crystals. (*b*) The basis vectors of GNNQQNY crystals indexed by *cctbx.small_cell* in this work are displayed in reciprocal space. a*, b* and c* are displayed in red, green and blue, respectively. Axes are in units of reciprocal ångströms. Two views of the same set of vectors are displayed from different angles. Needle crystals in the injection stream tend to align along the x* axis, which is orthogonal to the beam. The real-space *b* axis corresponds to the length of the needle crystals and is coaxial with the direction of the hydrogen bonds formed between strands of the β-sheet.

The completeness for this set is 89% to 2.5 Å resolution, with an overall redundancy of 10.5 (Table 2 ▶). One likely reason for the low completeness is the natural orientation of the crystals in the crystal-injection stream. The thin needles (Fig. 5 ▶ *a*) tend to align in the liquid jet, which limits the available sampling of reciprocal space. To test this hypothesis, we plotted the reciprocal basis vectors for all indexed images in Fig. 5 ▶(*b*). The b* axis (coinciding with the long direction of needle-crystal growth) shows a clear tendency for the crystals to preferentially align with the flow of the jet. We performed the same visualization with basis vectors derived from thermolysin crystals that had been analyzed using an XFEL source (Hattne *et al.*, 2014 ▶) and saw that the cloud of superimposed vectors was spherical, indicating they are randomly oriented in the stream (not shown).

**Figure 6:**
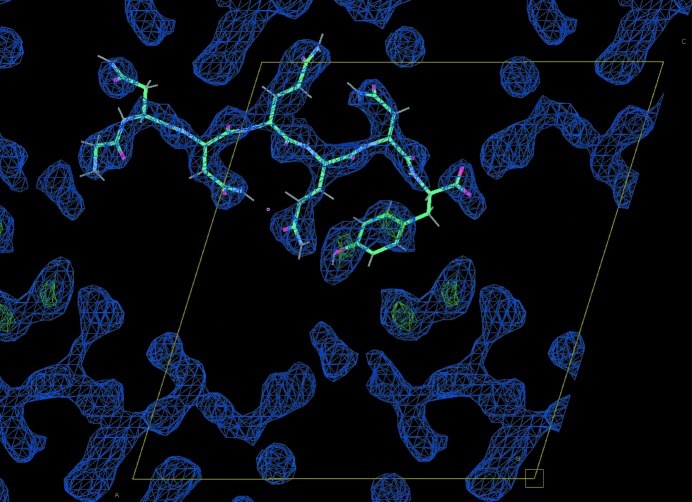
Refined GNNQQNY map from 232 images indexed by *cctbx.small_cell*. The GNNQQYNY peptide is shown in cyan. Blue density is the 2*F*_o_ − *F*_c_ map contoured at 1.5σ; *F*_o_ − *F*_c_ difference density is shown in red (negative) and green (positive) contoured at 3.0σ. The unit cell is drawn in yellow. This image was rendered using *Coot* (Emsley *et al.*, 2010 ▶).

Notwithstanding the biased orientation of the peptide crystals, the merged data did allow *Phaser* to produce an interpretable molecular-replacement solution using the published coordinates as a search model. A simple refinement using *phenix.refine* (Adams *et al.*, 2010 ▶) was performed starting from the *Phaser* solution. The resulting map shows features consistent with the peptide, and potentially different locations for water molecules (Fig. 6 ▶). The high *R*_work_ (34.4%) and *R*_free_ (41.5%) of these data are expected given the small amount of data merged. To confirm that the data set contains meaningful structural information, we performed three controls (see Supplementary Figs. S4 and S5). Firstly, we rotated by 90° and translated the molecular-replacement model to an incorrect location and passed it to *Phaser* for molecular replacement (Supplementary Fig. S4, magenta peptide). *Phaser* was able to place the molecule back into an orientation matching the published orientation, within tolerances on the *a* and *c* axes that match the difference in unit-cell sizes between this work and the published structure (see Figure S4, noting that the choice of *b* axis origin in this monoclinic point group is arbitrary). Secondly, as a negative control, we repeated this process but first shuffled the intensities in the merged data set. Here, *Phaser* was not able to recover the correct orientation of the peptide. Even if the initial model was already in the correct orientation before MR was attempted, *Phaser* could not find the correct solution (not shown). Finally, we generated a map in which we used intensities from the shuffled data set and calculated phases from the refined GNNQQNY peptide. We compared this map with the map from the nonshuffled data (Supplementary Fig. S5). The shuffled map is considerably noisier and less connected. Together, these are strong indicators of detectable signal from the *cctbx.small_cell* indexed data even when limited to a small number of indexable images.

## 4. Discussion   

While XFELs provide new avenues of biological investigation regarding small peptides, data-processing challenges continue to be discovered. Without rotational information, the sparseness of the GNNQQNY diffraction patterns renders them intractable using conventional indexing algorithms. We have developed a new set of indexing techniques using a synthesis of powder-diffraction methods and classic computer-science approaches that relates the indices of a diffraction pattern to nodes in a graph and resolves indexing ambiguities by determining a maximum clique of that graph. For practical use, a vastly greater quantity of data must be processed than presented here, which is expected to improve the quality of the statistics of the data and increase the completeness. The ability to correctly identify an MR solution, however, validates the potential of these algorithms in indexing these problematic crystallographic data.

As new crystal forms of biologically relevant peptides are discovered, we hope that these techniques will enable *de novo* structure solution of XFEL diffraction data collected from these crystals. This is an ambitious goal. Beyond the practical issues of crystal orientation and data quantity, the two primary hurdles in reaching it will involve accurate merging of integrated intensities, accounting for scale factors and partiality, and solving phases either from molecular replacement or from heavy-atom derivatives. Further work in developing these algorithms for stills is in progress.

## Supplementary Material

Supplementary Figures.. DOI: 10.1107/S1399004714026145/dz5348sup1.pdf

## Figures and Tables

**Table 1:** Effect of misindexing a single reflection

	*a* ()	*b* ()	*c* ()	()	()	()
Powder cell	22.23	4.86	24.15	90	107.32	90
Misindexed spot 11 (0, 1, 1)	23.62	4.84	26.62	89.61	113.84	90.05
Correctly indexed spot 11 (1, 1, 0)	23.09	4.87	25.09	91.43	110.81	88.47

**Table 2:** Data-processing statistics Values in parentheses are for the highest resolution bin.

Data collection	GNNQQNY
Wavelength ()	1.454 0.001
Space group	*P*2_1_
Unit-cell parameters (powder)†	
*a* ()	22.23 0.2
*b* ()	4.86
*c* ()	24.15 0.2
()	90.00
()	107.32
()	90.00
Unit-cell parameters (derived)‡	
*a* ()	22.60 2.3
*b* ()	4.88 0.1
*c* ()	24.72 1.7
()	90.18 1.8
()	107.40 3.1
()	89.8 2.1
Resolution ()	23.052.50 (2.592.50)
Reflections in total	2290 (42)
*I*/(*I*)	16.7 (10.6)
Completeness (%)	89 (73)
Multiplicity	10.5 (1.6)
*R*_work_/*R*_free_ (%)	34.4/41.5

† Unit-cell parameters derived from the maximum-value composite powder pattern synthesized from 32178 XFEL images. We estimated the error for this calculation to be 1% (see main text for details).

‡ Average of unit cells calculated from 232 indexed GNNQQNY XFEL images. The unit-cell parameters of each individual pattern were computed from the indices and reciprocal-space coordinates of all indexed spots in that pattern.

## Appendix A Maximum cliques, *cctbx.small_cell* and the Bron–Kerbosch algorithm

*cctbx.small_cell* computes indices for reflections by creating a graph and finding the maximum clique of that graph. Each node represents a reflection, keyed by the reflection’s arbitrary unique ID, a candidate index and a symmetry operation that moves that reflection to the asymmetric unit of a reference spot. The edges of the graph represent an abstract ‘connectedness’, where two nodes are connected if the observed distance in reciprocal space between them, calculated from the diffraction image and the properties of the experiment (detector distance, wavelength *etc.*), matches the predicted distance between them based on the *hkl* values and the metrical matrix of the unit cell. In other words, two nodes are connected if their observed locations in reciprocal space match their predicted locations calculated from their candidate Miller indices.

The full graph will contain the same reflection multiple times with different Miller indices and candidate symmetry operations. Within the graph, there will be sets of connected nodes. Each clique, or set of nodes that all connect to each other, will represent a set of reflections that if assigned their candidate Miller indices will have been indexed consistent with the symmetry of the known crystal space group and consistent with the observed positions of the reflections on the image.

So far, the case where a clique is formed that contains the same reflection twice with two different candidate indices has not been discovered. The same powder ring index would have to have been duplicated in the clique with a different symmetry operator applied to each, or the reflection would need to have overlapped two powder rings. In either case, the predicted reflection locations would not allow both spot/index combinations to be connected to the rest of the clique. One will be substantially mismatched from the rest of the clique. Importantly, the opposite scenario, where the same index is assigned to multiple reflections in the same clique, can occur. This is likely to be owing to split spots or multiple lattices (see §[Sec sec2.6]2.6).

Once the graph has been built, the goal is to search it for the largest clique of nodes that represent a set of self-consistent indices. From this clique, the crystal orientation can be derived (see §[Sec sec2]2). An example clique is shown in Supplementary Fig. S6.

To solve the graph for the maximum clique, we use the Bron–Kerbosch algorithm (Cazals & Karande, 2008 ▶). Briefly, the algorithm uses a recursive backtracking technique to iterate through the graph and assign nodes to cliques. We further use a pivot, taking advantage of the fact that when querying a set of nodes to see if they are members of a given clique, we need only query if the pivot is in the clique or if one of its non-neighbors is, because if the pivot is in the clique then its non-neighbors cannot be. Before execution, the nodes are sorted in order of increasing degree (*i.e.* number of connections) and the choice of pivot in a given recursive function call is chosen to be the node with the highest degree. Once all possible cliques in the graph have been found, the resultant list of cliques is sorted and the largest one is the maximum clique. The indices can then be directly used to calculate basis vectors in concert with their locations in reciprocal space.
