# Supplementary material for: Indexing amyloid peptide diffraction from serial femtosecond crystallography: new algorithms for sparse patterns
Source: Acta Crystallogr D Biol Crystallogr. 2015 Jan 23;71(Pt 2):357–66. doi: 10.1107/S1399004714026145 (PMC4321489; doi:10.1107/S1399004714026145)
Supplement: Supplementary file 1 [file d-71-00357-sup1.pdf]

BIOLOGICAL CRYSTALLOGRAPHY

Volume 71 (2015)

Supporting information for article:

Indexing amyloid peptide diffraction from serial femtosecond crystallography: new algorithms for sparse patterns

Aaron S. Brewster, Michael R. Sawaya, Jose Rodriguez, Johan Hattne, Nathaniel Echols, Heather T. McFarlane, Duilio Cascio, Paul D. Adams, David S. Eisenberg and Nicholas K. Sauter

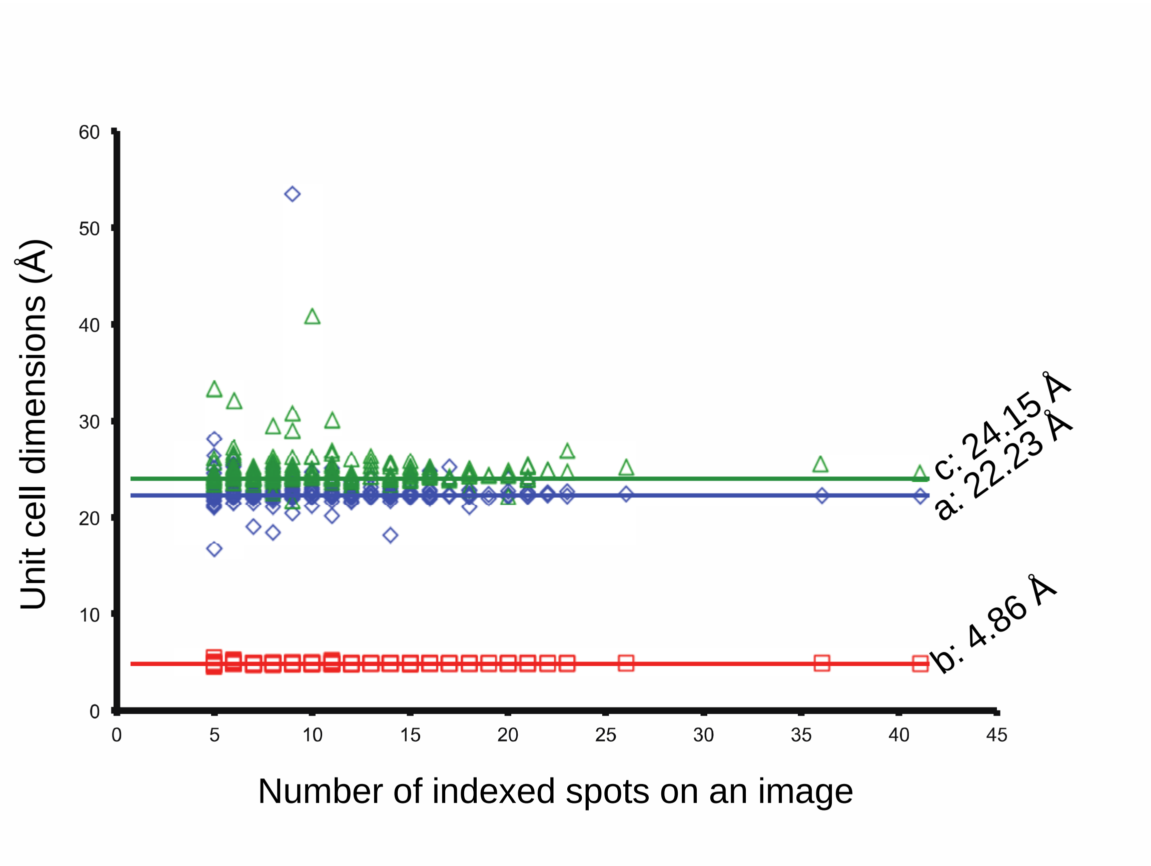

Figure S1: Effect of the number of indexed reflections on the derived unit cell parameters. 232 GNNQQNY images were indexed using *cctbx.small_cell*. For each image, rounds of refinement of the crystal orientation matrix were completed, adding reflections to the group of indices used to derive basis vectors after each round until no more reflections could be added. The final unit cell parameters derived from Equation 5 for each image are displayed. The unit cell parameters from indexing the maximum-value composite powder pattern are marked as blue, red, and green horizontal lines.

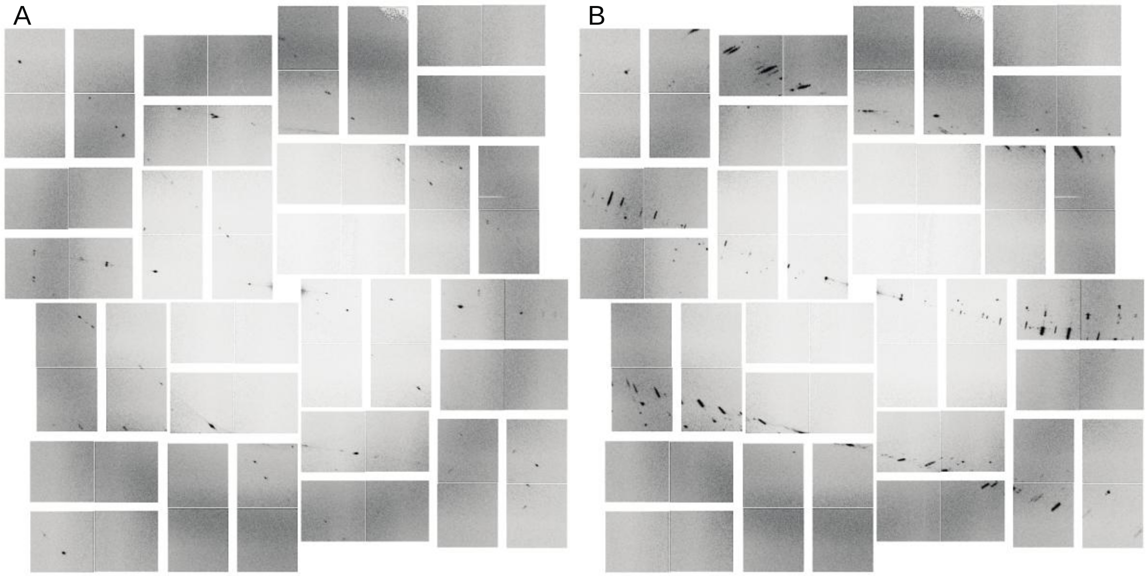

Figure S2: Examples of multiple (A) and split (B) GNNQQNY diffraction patterns.

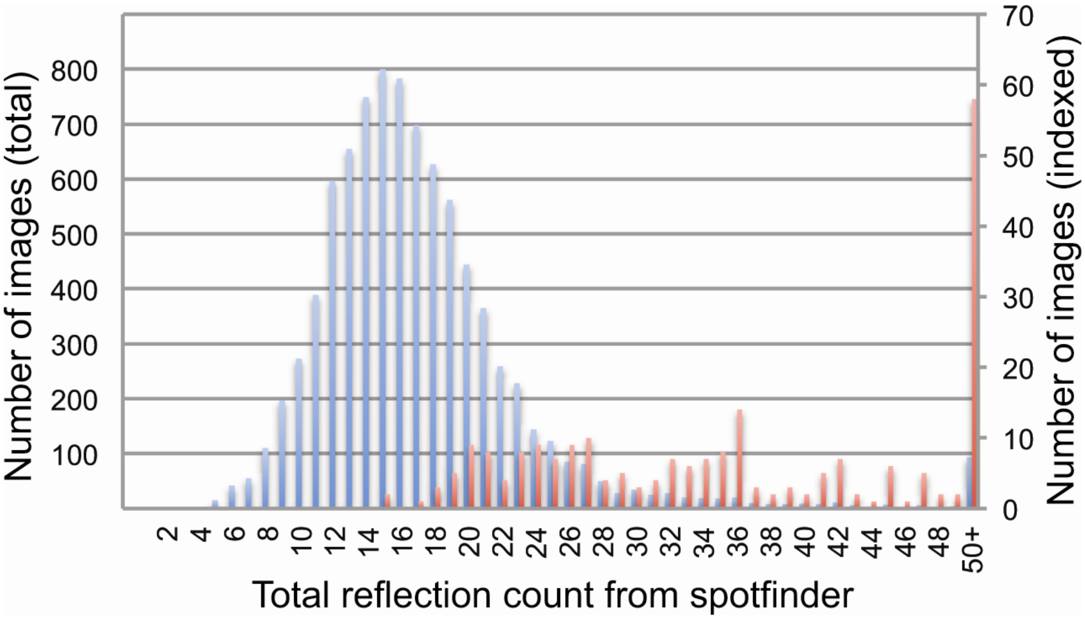

Figure S3: Histogram of total numbers of images vs. number of reflections found on those images. Left axis and blue columns: all images with potential signal (8704 overall). Right axis and red columns: indexed images (232 overall). Because our permissive spotfinder accepts images with small, weak clusters of pixels deemed as spots, the 8704 form a Gaussian distribution centered on 15 ‘spots’. Examining the unindexed images reveals few actual Bragg reflections, indicating this distribution corresponds to the noise inherent in the permissive spotfinder.

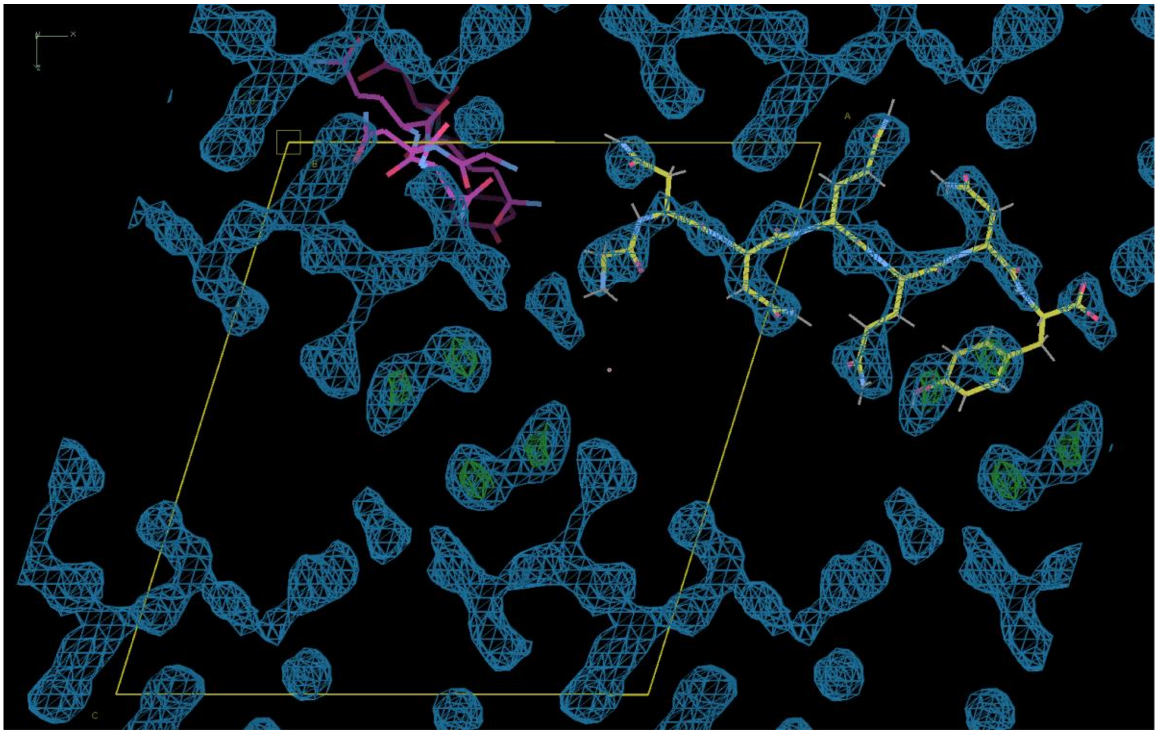

Figure S4: *Phaser* can re-orient and place a mis-oriented GNNQQNY peptide into its correct location. GNNQQNY $2F_o$-F_c electron density map from this work is displayed as in Figure 6, main text. Two peptides are displayed: the mis-oriented GNNQQNY peptide in magenta (translated and rotated orthogonally away from the correct placement) and the correctly placed peptide in yellow. *Phaser*, using the *cctbx.small_cell*-reduced dataset from this work, placed the yellow peptide in the orientation shown,. There is an origin shift in the *b* direction (not visible) that is allowed in the monoclinic $P2_1$ space group.

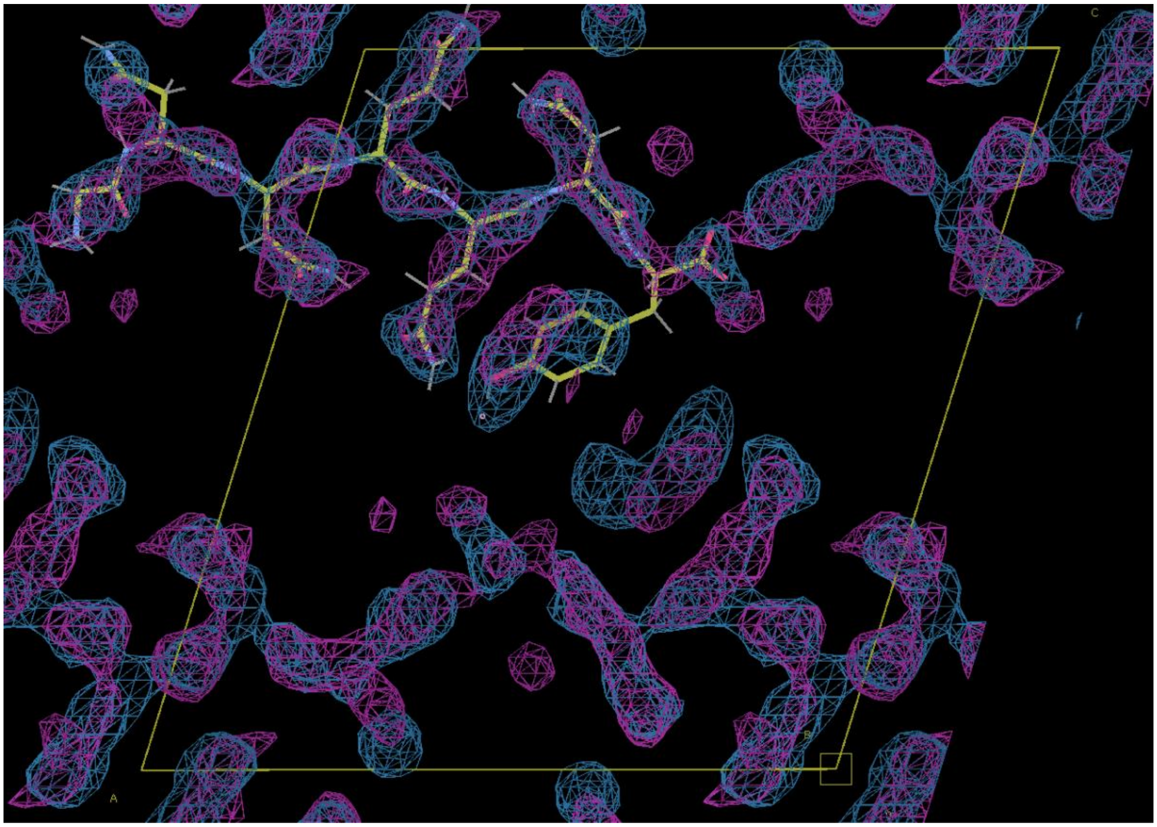

Figure S5: Shuffled intensities worsen the map. Cyan: refined GNNQQNY $2F_o$-F_c map displayed as in Figure 6 main text. Magenta: intensities from the same MTZ file were binned by resolution and shuffled randomly within these bins using *phenix*, and then a new map was generated using the model phases from the refined GNNQQNY structure. The $2F_o$-F_c map is displayed. Both maps are contoured at 1.5σ.

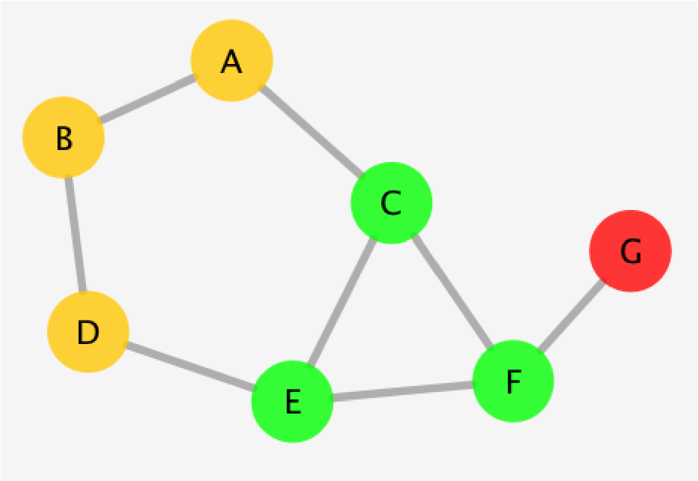

Figure S6: Example clique. Nodes are colored by degree (number of connections), as in Figure 4c, with green being many connections and red being one. {C, E, F} is the graph's maximum clique; each node in that clique is connected to the others in that clique, and other cliques, such as {B, D}, are smaller.
